# Examining the Feasibility and Acceptability of Valuing the Arabic Version of SF-6D in a Lebanese Population

**DOI:** 10.3390/ijerph17031037

**Published:** 2020-02-06

**Authors:** Samer A. Kharroubi, Yara Beyh, Marwa Diab El Harake, Dalia Dawoud, Donna Rowen, John Brazier

**Affiliations:** 1 Department of Nutrition and Food Sciences, Faculty of Agricultural and Food Sciences, American University of Beirut, P.O. Box 11-0236, Riad El Solh, Beirut 1107-2020, Lebanon; yara@sbeyh.com (Y.B.); md106@aub.edu.lb (M.D.E.H.); 2 School of Health and Related Research, The University of Sheffield, Regent Court, 30 Regent Street, Sheffield S1 4DA, UK; d.rowen@sheffield.ac.uk (D.R.); j.e.brazier@sheffield.ac.uk (J.B.); 3 Clinical Pharmacy Department, Faculty of Pharmacy, Cairo University, Cairo 11562, Egypt; ddawoud@hotmail.com

**Keywords:** Lebanese, SF-6D, preference-based measure, reliability, validation, standard gamble

## Abstract

*Objectives:* The SF-6D is a preference-based measure of health developed to generate utility values from the SF-36. The aim of this pilot study was to examine the feasibility and acceptability of using the standard gamble (SG) technique to generate preference-based values for the Arabic version of SF-6D in a Lebanese population. *Methods:* The SF-6D was translated into Arabic using forward and backward translations. Forty-nine states defined by the SF-6D were selected using an orthogonal design and grouped into seven sets. A gender-occupation stratified sample of 126 Lebanese adults from the American University of Beirut were recruited to value seven states and the pits using SG. The sample size is appropriate for a pilot study, but smaller than the sample required for a full valuation study. Both interviewers and interviewees reported their understanding and effort levels in the SG tasks. Mean and individual level multivariate regression models were fitted to estimate preference weights for all SF-6D states. The models were compared with those estimated in the UK. *Results:* Interviewers reported few problems in completing SG tasks (0.8% with a lot of problems) and good respondent understanding (5.6% with little effort and concentration), and 25% of respondents reported the SG task was difficult. A total of 992 SG valuations were useable for econometric modeling. There was no significant change in the test–retest values from 21 subjects. The mean absolute errors in the mean and individual level models were 0.036 and 0.050, respectively, both of which were lower than the UK results. The random effects model adequately predicts the SG values, with the worst state having a value of 0.322 compared to 0.271 in the UK. *Conclusion:* This pilot confirmed that it was feasible and acceptable to generate preference values with the SG method for the Arabic SF-6D in a Lebanese population. However, further work is needed to extend this to a more representative population, and to explore why no utility values below zero were observed.

## 1. Introduction

The fast growth medical technologies and treatments increasingly requires cost-utility analyses (CUA) and cost-effectiveness analyses (CEA) to decide on the optimal treatment for every health condition [1]. Agencies that advise on reimbursement such as the National Institute for Health and Care Excellence (NICE) commonly require a health-related quality of life (HRQoL) outcomes using quality adjusted life years (QALYs) from preference-based measure questionnaires as part of the decision-making process [2,3]. In order to generate QALYs, there must be a valuation for HRQoL on the 1–0 full health–death scale through eliciting preferences from the general population by setting full health to 1 and death to 0 on the scale [4,5].

To date, a large number of preference-based measures exist, including the generic EQ-5D [6], EQ-5D-5L [7], Healthy Utilities Index 2 (HUI2) and HUI3 [8,9], Assessment of quality of life (AQoL) [10], Quality of Well-Being scale (QWB) [11], and the SF-6D [12], in addition there are a growing number of condition-specific preference-based measures [13]. All of these preference-based measures of health have been valued in their countries of origin, and there is increased interest in obtaining values from other countries, for example [14,15,16,17,18,19,20,21,22], among many others.

The SF-36 [23,24] served as the base for the SF-6D used in several valuation studies. Previously, a scoring algorithm for the SF-6D has been derived from the general UK population using the standard gamble (SG) technique [12]. This has been used to elicit values for several countries, including China [19], Japan [20], Hong Kong [21], Brazil [22], Portugal [23], and Australia [24]. There were significant differences between UK values and the other countries, which suggested cultural differences in values. It is likely that significant differences in the preferences for different health states may also exist in a Lebanese population relative to other populations. To the best of our knowledge, there have been no studies in any Middle Eastern country to elicit valuations for the SF-6D health states.

However, a few steps must be taken before applying the SF-6D to the Lebanese population. First, it is imperative to confirm the ability to generate a Lebanese preference-based valuation for the multi-dimensional SF-6D health states using preference elicitation tasks. Second, it is compulsory to check if a valuation of the representative population can be used to produce a scoring algorithm to generate utility values for all possible SF-6D states. Since most studies using SG have been conducted on Western populations, little is known about its feasibility, validity, and reliability in Middle Eastern populations including the Lebanese population.

Thus, the aim of this pilot study is to examine the feasibility and acceptability of valuing the Arabic SF-6D in a Lebanese population using the SG method. If the results are positive, preference-based measures of health such as the SF-6D could be valued by the Lebanese population to generate a definitive value set for Lebanon. This may enable the inclusion of the Lebanese population in global and multi-ethnic pharmacoeconomic evaluation studies.

In the following sections, we describe the methods of the SF-6D valuation survey and the data collection process. The modeling of the valuation data is also outlined. Then, we present our findings in Section 3 and finish with a discussion of the results, their implications, and briefly consider the possible future studies.

## 2. Methods

### 2.1. The SF-6D

The SF-6D is derived from the SF-36. It is composed of six health dimensions, including physical functioning, role limitation, social functioning, bodily pain, mental health and vitality, each having between four and six levels [12]. Defining a health state requires choosing a level from each dimension, hence creating 18,000 possible combinations. Since every possible health state is described by six digits, from 1 to 6, the perfect health state (full health) is indicated by the combination 111,111, whereas the “pits” (worst health state) is indicated by 645,655.

### 2.2. Subjects

Lebanese adults aged between 18 and 70 years old were recruited at the American University of Beirut (AUB), stratified based on gender (male/female) and on occupation (faculty/staff and employees/students). Potential participants were contacted by phone and/or email to schedule an interview session. However, those who could not be reached after two attempts and those unwilling to participate were excluded from the study.

As this was a feasibility study, a formal sample size calculation was not undertaken. Previous experiences with the SF-6D have shown that 15 observations per health state are adequate to estimate a new model [19]. Hence, a total of 126 people, 21 in each of the six gender-occupation groups, were interviewed out of 170 initially contacted potential participants, thus giving a response rate of 74%. Each one of the seven sets of health states (see below for further details) was valued by three respondents from every group (gender-occupation) for a total of 18 valuations per health state. In order to assess the reliability of the questionnaire, a random sample of 21 participants across all six groups was interviewed a second time 2–4 weeks after the initial interview.

### 2.3. Data Collection Procedure

An Arabic version of the original SF-6D Health Survey was developed by forward and backward translations using professional translators. The latter has been done in collaboration with a team in Egypt and the United Arab Emirates (UAE) [25], for which the English equivalence has been approved by the developers Brazier and Kharroubi. Given that the SF-6D is known to be an elaborated descriptive system, with 18,000 possible outcomes, a sample of 49 health states was generated using the orthoplan procedure in SPSS (SPSS Inc., Chicago, IL, USA). For the sake of future comparison, the 49 health states chosen were the same used in the feasibility study of Chinese SF-6D valuation by Lam et al. [19], and which included every level of every dimension at least once. Those states were then distributed over seven sets, each containing seven health states each represented by a six-digit number, where each digit denotes a level from the SF-6D dimensions in the following sequence: Physical functioning (PF), role limitation (RL), social functioning (SF), pain, mental health (MH), and vitality (VIT). In addition, each respondent valued “pits” (worst health state).

Interview sessions took place between late January 2019 and early March 2019. The interview officially started after briefly explaining the study to the participants and obtaining their written consent. The sets of health states were used in a rotational manner to reduce the interviewer learning effect. The interview session followed a certain sequence of events, where the subject was asked to: (1) Answer the Arabic version of the SF-6D; (2) rank eight health states (the set of seven health states and the “pits” state); (3) value the seven health states and the pits ranked using the SG technique used by Brazier et al. [12] in a random order to reduce the bias effect that could arise from the order of the states; (4) provide some information about their demographics; and (5) fill in an evaluation survey about the interview. The study has been ethically approved by the Institutional Review Board (IRB) at the AUB.

The interview protocol was analogous to the one applied in the UK valuation study [12]. The aim was to allow fair comparison across the two valuation studies. Each respondent was asked to rank and value eight health states using the McMaster ‘ping pong’ variant of the SG [26]. The SG technique asked the respondents to value seven of the eight SF-6D health states against the perfect health state and the “pits” state. Respondents were then asked in the eighth SG question to value “pits”. Depending on whether they thought this state was better or worse than death they would be asked to consider one of the following choices: (i) The certain prospect of being in the “pits” state and the uncertain prospect of perfect health or immediate death; or (ii) the certain prospect of death and the uncertain prospect of perfect health or the “pits” state. The chances of the best outcome occurring is varied until the respondent is indifferent between the certain and uncertain prospects. The negative of the indifference probability of the best outcome is used to value states worse than death, having the effect of bounding negative values at −1 [27]. Then, the other seven health states were chained onto the zero to one scale, where 0 is given to states perceived equivalent to being dead, and 1 is given to perfect health [12]. Having valued the “pits” state (P), the seven intermediate SF-6D health state valuations (SG) are adjusted using the formula SG + (1 − SG)*P, where the best SF-6D state is 1 and death 0, for use in the modelling.

The interview material was in Arabic and the interviews were conducted by a trained interviewer, who after the interview reported their views on the understanding, effort, and concentration of the subject. The respondent also reported how they found the SG tasks.

### 2.4. Patient and Public Involvement

Patients or the public were not involved in the design, or conduct, or reporting, or dissemination of the research.

### 2.5. Data Analysis and Outcome Measures

This study evaluated different aspects of the Arabic SF-6D. First, the feasibility of the health survey was assessed by (1) the completion rate of the interviews; (2) percentage of states with useable values; (3) interview’s duration; (4) respondent understanding, effort, and concentration as reported by the interviewer; and (5) respondents own rating of how they found the SG tasks including their effort, frustration, and boredom.

Data were considered unusable if the results obtained from the respondents met any one of the following three conditions: (1) All health states had the same valuation; (2) less than two health states were valued; and (3) pits state was not valued. The valuation of the pits state was essential in order to chain the respondents’ health state on the full health-death scale, where full health had a value of 1, dead had a value of zero, and any negative value was bounded by −1. These adjusted SG values form the dependent variable (y) in the models discussed below.

The test–retest reliability of the survey was assessed by analyzing the results obtained from the 21 re-interviewed subjects using the mean difference between test and retest results (statistical significance tested by paired *t*-test), and intraclass correlation (ICC) calculated using the two-way mixed effects model where respondents’ effects were considered as random and interviewers’ effects were fixed. As for the validity of applying standard modelling techniques, this was assessed by fitting the models to Lebanese SG data and comparing predictive ability and consistency of the model coefficients with the results of the UK SF-6D.

To understand the size and potential importance of differences between the UK value set and this Lebanese population we also compared the distribution of values, mean health state values for the 39 common states, and their intra class correlation. We also compared the ranking of the coefficients from the models.

### 2.6. Modelling

The modelling methods followed the same methods as the UK study [12]. Models have been estimated at the aggregate level; that is, the explanatory variables were used to estimate the mean value given to each of the states by the respondents that valued them (the mean level model). Models have also been estimated at the individual level that takes into account the variation both within and between respondents using a random effects (RE) model.

The general model for health state valuations is:

(1) $$y_{ij}=g\left(\beta^{\prime }x_{ij}+\theta^{\prime }r_{ij}+\delta^{\prime }z_{ij}\right)+\varepsilon_{ij}$$

where *i* = 1, 2, …, *n_j_* represents individual health state values and *j* = 1, 2, …, *m*, $y_{ij}$ represents individual respondents, *g* is a function specifying the appropriate form, and $\varepsilon_{ij}$ is an error term, whose properties depend on the assumptions of the model [12]. The dependent variable, $y_{ij}$, is the adjusted SG score for health state *i* valued by respondent *j*, **x** is a vector of binary dummy variables for each *λ* of dimension *δ* of the descriptive system, where the best level of each dimension represents the baseline for that dimension. For example, *x*_32_ denotes dimension *δ* = 3 (social functioning), level *λ* = 2 (health limits social activities a little of the time). For any given health state, *x_δλ_* is defined as:

*x_δλ_* = 1 if, for this state, dimension *δ* is at level λ
*x_δλ_* = 0 if, for this state, dimension *δ* is not at level *λ*

In all, there are 25 of these terms, hence, for a simple linear model, the intercept represents state 111,111, and summing the coefficients of the ‘on’ dummies derives the value of all other states. The *r* term is a vector of terms to account for interactions between the levels of different attributes. However, given the small sample size, we did not look at interaction terms here. Finally, **z** is a vector of respondent level characteristics such as age, sex, or socio-economic factors.

Mean level models were estimated using the ordinary least squares (OLS) and random and fixed effects models were also estimated using generalized least square (GLS) and maximum likelihood estimation in order to take into account repeated observations for each individual [12]. For the random effects (RE) model the error term, $\varepsilon_{ij}$, is subdivided as follows:

(2) $$u_{j}+e_{ij}$$

where $u_{j}$ represents the individual random effect, assumed to be random across individual respondents, and $e_{ij}$ represents the random error term for the health state valuation *i* of individual *j*.

The models were evaluated considering the following criteria: (1) Inconsistencies in the estimated coefficients, as the coefficients of dummy variables representing each level of SF-6D are expected to be negative and increasing in absolute size as the level of severity increases (amongst coefficients with statistical significance); (2) adjusted R^2^, mean absolute error, and the proportion of predictions outside 0.05 (% absolute error > 0.05) and 0.10 (% absolute error > 0.10) ranges on either side of the observed value. Predictions were further tested in terms of bias (*t*-test). Analysis was performed using SPSS version 24.0 (SPSS Inc., Chicago, IL, USA) (Statistical Package for Social Sciences. Available from: http://www.spss.com/software/) and R 2.9.1 (R Development Core Team, Vienna, Austria) (R: A language and environment for statistical computing. R Foundation for Statistical Computing. Available from: www.R-project.org).

## 3. Results

### 3.1. Participants

One hundred and twenty-six participants were recruited from AUB and belonged to either one of the three following categories: (1) Faculty, (2) staff, and (3) students. The mean age of the participants was 32.45 years, which is very close to the mean age of the Lebanese general adult population (31 years) [28]. The gender distribution (male/female) of the subjects (50.8%/49.2%) was in line with that of the general population (50.2%/49.8%). However, 88.7% of the participants hold a degree or above, which is very far from the descriptive of the general population (13.8%), and 71.3% have a total household income higher than 2200 USD. The discrepancy in educational level and the high total household income are due to our sample population being recruited from an educational institution. As for the housing, a large proportion of respondents (41.9%) live with their parents, since one third of our data was collected from students. The marital status is consistent with that of the general population, since 63.7% of the Lebanese are listed as single. More information about the sociodemographic characteristics of the interviewed population is available in Table 1.

### 3.2. Feasibility and Acceptability

The 126 recruited participants completed all parts of the questionnaire, thus providing a 100% completion rate. Two subjects out of the 126 participants gave the same valuation for all eight valued health states, including the pits, and were excluded from the data analysis. No respondents were excluded for failing to value two or more health states or for failing to value the pits state. The mean time for completing the whole interview was 26.98 min (SD 8.62, ranged from 11 to 70).

Table 2 shows the interviewer and respondent evaluations on the process of the ranking and SG exercises. According to interviewers’ evaluations, the vast majority of respondents had no problems or only some problems performing and concentrating on the ranking task (over 99% and 94.4%, respectively) and SG (over 99% and 93.5%). In regard to the respondents’ evaluations, almost all of the respondents (92.7%) mentioned trying their best in answering the questionnaire. Half of the respondents (50.0%) said that they considered three or more dimensions in the SG decision indicating the majority were not lexicographic in their preferences. None of respondents found the task very difficult and none thought the quality of their answers was poor. The process was acceptable to most subjects with 75.0% evaluating the ranking and SG tasks as easy or neutral and 79.0% reporting no degree of irritation or boredom during any of the ranking or SG exercises.

### 3.3. Test–Retest Reliability

Three to four weeks after the first interview, a random pool of 21 respondents were selected for a repeat interview, in order to check the reliability of the questionnaire. The ranking of the best health state card as the top was consistent in both interviews for all 21 respondents. On the other hand, six (28.6%) respondents reversed the order of the pits and severe cards between the first and second interviews (two ranked a severe card the lowest in the first interview but the pits health state lowest in the second interview; and four ranked the pits health state the lowest in the first interview but a severe card the lowest in the second interview). There were 168 paired health state values for the assessment of the test–retest reliability. The mean difference of SG valuations between baseline and post-test was 0.0092 (95% CI −0.02, 0.04), which was not statistically significant by the paired *t*-test (*t* = 0.549, *p* = 0.583). The ICC of SG valuations between baseline and post-test was 0.667 (95% CI 0.55, 0.75), which was almost in line with the standard of 0.7 for group comparison [29].

### 3.4. SF-6D Valuation

Each of the 126 subjects valued seven health states from the SF-6D in addition to “pits”, resulting in a total of 1008 health state valuations (882 observations for the health states and 126 for “pits”). The number of observations was evenly distributed across the 49 health states selected by orthoplan using SPSS. All 126 participants were able to value the seven health states in addition to “pits”, however, two of them provided the same valuation for all eight health states (including “pits”). Therefore, in total, we had 992 (98.4%) useable observations (868 observations for the health states and 124 for “pits”). Table 3 shows the mean with SD, median, minimum, maximum values and the number of usable values of the 49 SF-6D health states valued in the sample and “pits”. These results were compared to the valuations from the UK study. However, it is important to note that some health states valued in this pilot study were not part of the original UK study (health states 124,125, 135,312, 212,145, 221,452, 334,521, 425,131, 432,621, 523,551, 534,113, and 611,221), and hence their appropriate cells in Table 3 were left empty.

It can be seen that the observed values for the “pits” state (645,655) in this pilot ranged between 0.100 and 0.750, with a mean value of 0.322 (±0.190), whereas in the UK study, its values ranged between −0.980 and 0.980 with a mean of 0.213 (±0.428). As for the best health state (211,111), its observed valuations ranged between 0.820 and 0.960, with a mean value of 0.890 (±0.042), whereas in the UK study they ranged between 0.190 and 1.000 with a mean value of 0.778 (±0.276). For an example of a moderate state, the observed mean value for state 142,154 is 0.791 (±0.159), with a range of 0.370–0.920, whereas in the UK study, its mean value is 0.513 (±0.378), with a range of 0.280–0.950. For 36 out of the 39 common states the mean health state values from the Lebanese sample exceeded the equivalent UK values by an average of 0.135 (±0.123). The overall level of agreement between UK and Lebanese mean health state values was not high with an ICC of 0.172 (95% CI −0.087, 0.438).

**Figure 1:**
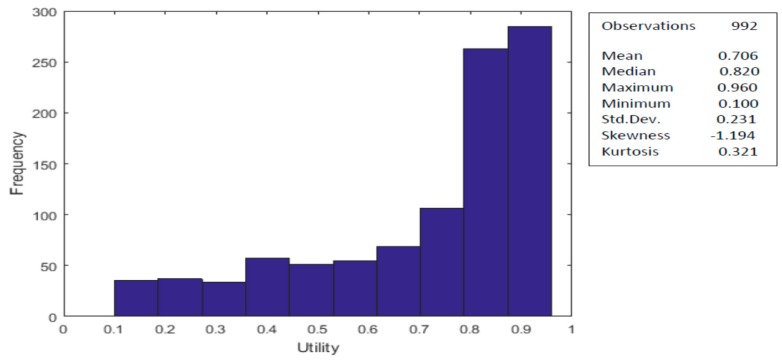
Histogram and descriptive statistics for the adjusted health state valuations (*n* = 992).

The left skewness in elicited utility values at the individual level is shown in the histogram for the 992 individual health states values in Figure 1. There are no negative values observed in the Lebanese sample. However, a large proportion of the values were above 0.9 (19%), as also observed in the UK (23%). There were no utility values at 1.0, which indicates that all participants were willing to risk a worse health state to have a chance for a better state.

### 3.5. Modelling

The models used for the analysis were random effect (RE) models at the individual level and the ordinary least square (OLS) model at the aggregate (using the mean values of the 50 valued health states) level. In both models, the constant was restricted to unity. The results of the obtained beta coefficients estimated for each level in every dimension, model predictive ability (MAE and number of absolute errors greater than 0.05 or 0.10), and the number of inconsistent preference-based coefficients are presented in Table 4. Our results are compared to those of the UK valuation study by Brazier et al. [12]. Coefficients found to be significantly different from zero at α > 0.05 are marked in bold. For the RE model, 17 out of the 25 parameters were significant. However, at α > 0.10, an additional parameter (VIT4) became significant, meaning a total of 18 out of 25 parameters were significant. The parameter estimates for physical functioning and social functioning were very similar to those of the UK. For instance, the coefficient for PF6 was −0.173 compared to 0.160 in the UK, and that of SF5 was −0.116 to −0.109. However, there were marked differences in the coefficients for pain across all levels with level 6 scoring −0.093 in the Lebanon compared to −0.178 in the UK. There were smaller but nonetheless potentially important differences in the coefficients for the other dimensions. The order of the decrements in the Lebanese model resulted in a ranking of PF with the largest, followed by RL, SF, Pain, MH, and VIT. This contrasts with the UK that also had PF first, but this was followed by Pain, MH, SF, VIT, and RL.

All coefficients in the UK study were negative in the RE model [12]. In our study, we had two parameters showing positive coefficients (PAIN3 and VIT2), both of which are insignificant. The MAE in the RE model for Lebanon was better than that of UK; 0.050 compared to 0.078 in the UK. Two significant inconsistent coefficients were found, where the estimated effect decreases from level 2 to level 3 for the physical functioning (i.e., PF2 (−0.061) vs. PF3 (−0.056)) and level 2 to level 3 for the role limitations (i.e., RL2 (−0.057) vs. RL3 (−0.039)) in the RE model. However, the UK model had four such inconsistencies. 

As for the OLS mean model, the UK study observed 23 significant parameters while we observed 14 out of 25 parameters to be significant at α > 0.05 and at α > 0.10, an additional parameter (RL4) became significant, for a total of 15 out of 25 parameters. This smaller number may have been a result of a much smaller sample size. The UK mean model had two positive coefficients (PF3 and PAIN2), whereas in the Lebanese mean model VIT3 was positive. The MAE for Lebanon was smaller than that of UK, 0.036 and 0.074, respectively, as is the case with the RE model. Again, there were important differences in the parameter coefficients estimated from the OLS mean model for Lebanon compared to those of the UK. This time the ordering of decrements was PF followed by SF, Pain, MH, RL, and VIT. The UK mean model places pain at the top, followed by MH, PF, VIT, SF, and RL. This suggests there may be major differences in the relative weights for these dimensions.

**Figure 2:**
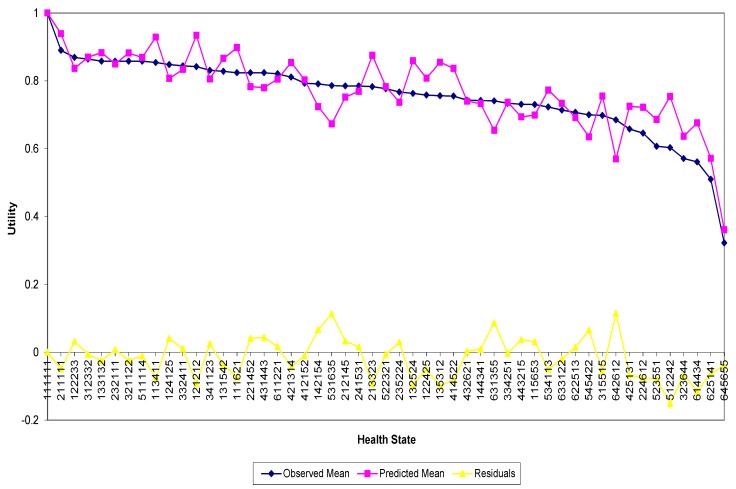
Actual and predicted health state valuations for the RE model.

Overall the models on the Lebanese valuation data had good performance. Two significant inconsistent coefficients were found, where the estimated effect decreases from level 2 to level 3 for the role limitations (i.e., RL2 (−0.049) vs. RL3 (−0.004)) and level 4 to level 5 for the mental health (i.e., MH4 (−0.098) vs. MH5 (−0.064)) in the OLS mean model. In comparison, the UK model had five such inconsistencies. The adjusted R^2^ of the OLS mean model for Lebanon was almost double that of UK, 0.950 and 0.508, respectively. Figure 2 presents the actual and predicted valuations for the RE model for the 49 valued health states and the pits. The RE model predicts the observed health state values quite well and in contrast to the UK model, does not seem to suffer from the tendency to over predict at low health state values (i.e., poor health states).

Finally, there is a key finding from the models that is worth mentioning. Namely, for the Lebanese sample, there is almost no discrimination in preference-based coefficients as a function of severity for either the pain or vitality dimensions. For both of these dimensions, coefficients for all but the most severe level of each are not statistically significant (and having small magnitude), and even the most severe level of vitality is not statistically significant for the aggregate model. In the results above, we pointed to the fact that there were fewer inconsistencies in magnitude of coefficients across dimension severity levels for the Lebanese sample than for the UK sample in the models, but we only focused on statistically significant coefficients in this count. Whether we use the actual values of non-significant coefficients, or if we just consider their values to be 0, there are many more inconsistencies for the Lebanese sample. We consider this finding in more detail in Section 4.

## 4. Discussion

Health state valuation is a relatively new research area in the Middle East, with two studies investigating the validity and reliability of the Arabic version of the EQ-5D-3L in Jordan and Saudi Arabia [30,31] and only one study focused on testing the feasibility of eliciting EQ-5D-5L values from a general public sample in the UAE [32]. The Arabic translation of EQ-5D-3L appeared to be valid and reliable in measuring the quality of life in Jordanian and Saudi people. In addition, results suggested that it is feasible to generate meaningful health-state values in the UAE and most of the respondents stated that their religious beliefs influenced their responses to the valuation tasks.

The results of this pilot study supported the feasibility and acceptability of using the SG method to generate health state utility values for the SF-6D in Lebanon, to generate QALYs, and hence to conduct cost utility analysis of health care interventions. The Lebanese SF-6D preference weights estimated here offer a method for producing utility values from existing SF-36 data. We believe that using SF-6D health state preference values from the Lebanese population to conduct cost-effectiveness studies in Lebanon is more appropriate than using values obtained in other countries. The results from our study sample were positive in a sense that preference-based measures of health such as the SF-6D could be adapted nationally to the Lebanese population who can then be included in global and multi-ethnic pharmacoeconomic studies.

After excluding unusable data from two participants, we had a completion rate of 98.4% which is much higher than that obtained in the UK where 36.8% of respondents were excluded from the data analysis [12]. This may be because we had a well-educated sample and only had two well-trained interviewers who made sure that respondents valued every health state. The Lebanese values were higher than those of the UK for 36 out of the 39 comparable states. Furthermore, the ordering of the dimension coefficients indicates a higher weight is given to PF, RL, and SF compared to pain and MH than the UK population. This indicates important possible differences in health preferences between the two cultures.

The mean health states for the 49 valued states were broadly consistent with the severity of the health state. This means that the scoring of the health state decreased with the increasing number of dimensions with severe levels in that state i.e., the misery score, which is the sum of all the severity levels of each dimension (e.g., the misery score for state 511,114 = 5 + 1 + 1 + 1 + 1 + 4 = 13). For instance, health state 511,114 had a mean value of 0.858 while state 512,242 had a value of 0.603.

The performance of the Lebanese models was compared to that of the UK model, and they both had good comparative performance relative to the UK models with MAE of 0.036 compared to 0.074 and adjusted R^2^ of 0.950 compared to 0.508 for the OLS mean model; and MAE of 0.050 and 0.078, respectively, for the RE model. These results support the validity of the preference-based valuation by SG of the SF-6D in a Lebanese population for the generation of scoring algorithms applicable to the Lebanese population. 

There were two significant inconsistencies in the estimated significant coefficients, where the estimated effect decreases from level 2 to level 3 for the physical functioning (i.e., PF2 (−0.061) vs. PF3 (−0.056)) and level 2 to level 3 for the role limitations (i.e., RL2 (−0.057) vs. RL3 (−0.039)) in the RE model. However, the UK model had four such inconsistencies. Two more significant inconsistent coefficients were found, where the estimated effect decreases from level 2 to level 3 for the role limitations (i.e., RL2 (−0.049) vs. RL3 (−0.004)) and level 4 to level 5 for the mental health (i.e., MH4 (−0.098) vs. MH5 (−0.064)) in the OLS mean model, whereas the UK model had five such inconsistencies. These results will further support the validity and quality of the data from the Lebanese population. These results are promising given the relatively smaller size of the Lebanese sample compared to the UK.

The models show that two of the six dimensions, pain and vitality, have small and insignificant coefficients for all severity levels with the exception of the worst level (s), and for vitality there are no significant coefficients for the mean level model. While it would be expected that values on some dimensions may differ across cultures (e.g., one would expect that the importance of social functioning could be different across different cultures, we would expect pain severity in particular to impact on health state preferences. This raises the question whether this finding would be observed in a larger sample of respondents, and whether this finding would be observed using a valuation study including a larger number of health states. However, if this result is replicated in a larger study this raises the question as to whether pain and vitality are relevant for inclusion in a preference-based measure in Lebanon if the Lebanese population do not state that milder and moderate problems with these dimensions impact on their utility. This is an important issue and is the subject of further work.

Limitations of this study include the use of a small sample of 126 people. This is much smaller than the UK study which involved 611 people, so it may limit the generalizability of the preference values found. More sophisticated models could be tested with data obtained from a larger sample of health states (Kharroubi et al. [33,34,35,36,37,38,39]). However, this study aims at testing the feasibility and acceptability of the use of SG to value the SF-6D in Lebanon, in order to proceed with a larger valuation study involving a larger number of participants. Further research is underway to assess this. In particular, ongoing valuation study for a sample of 249 health states defined by the SF-6D by a nationally representative sample of 577 participants matched with the national proportionate of gender and age category from all Lebanese governorates using standard gamble has preliminary results that are very promising. Upon completion, this study would be the first valuation study of the SF-6D in the Middle East, and therefore, neighbouring countries would benefit from this value set until similar studies are conducted in the region.

Whilst previous studies have found a relatively low number of utility values below zero elicited using the SG technique (for example the UK valuation of SF-6D using SG found 7% of responses were below zero [12]), the lack of utility values below zero is surprising. One possibility is that this was due to the small sample size, but a small number of utility values might still be expected with the sample size analysed here. There are many potential reasons why no values below zero have been observed, including attitudes to risk, characteristics of the particular survey sample, and the perceived severity of the states. There could be cultural or religious reasons why participants are not willing to say a health state is worse than being dead. For these reasons the interviewers may have been reluctant to move to the task for states worse than dead. Future research is recommended to explore this further since this is the first valuation study conducted in the Lebanese population.

An additional concern is around the understanding and concentration of study participants. Whilst the study participants report that they tried their best to answer (92.7%) and few felt bored or irritated (21.0%) only 50% of participants considered three or more of the dimensions in making their choices, and 25.0% of participants found the level of the task difficult. The level of difficulty of the task is to be expected given the complexity of the task. The fact that 50% of people considered only two or fewer dimensions when making their choices may reflect a simplifying heuristic observed in discrete choice experiments too whereby participants may have focused on a small number of dimensions to make the tasks easier to complete. This question is rarely asked in valuation surveys and so it is not possible to say whether this is unusual. However, the interviewer reporting of participant effort and concentration and problems in performing the tasks indicates that only a small number of participants had a lot of problems with the SG task (0.8%), or were reported to have had little effort and concentration (6.5%).

Our study sample was recruited from AUB, hence, the majority of our respondents were well educated (88.7% hold a degree or above). This may lead to a concern about whether SG is feasible and acceptable for use with people with low education levels, because SG requires the respondent to think in abstract terms of probability. Furthermore, the small number of health states valued could impact on the accuracy of the econometric modelling. Overall, the generated SF-6D preference-based coefficients from this pilot study should not be regarded as necessarily representative of the general population of Lebanon. Further studies with a larger and more representative sample from the general population are required to generate a definitive SF-6D value set for the Lebanese population.

## 5. Conclusions

This study has demonstrated that generating a scoring algorithm for the SF-6D for the Lebanese population using the SG technique is overall feasible and acceptable. The performance of the econometric models derived from the Lebanese data compared favorably to the UK study, particularly given the smaller sample size. Given the overall encouraging nature of the results, this suggests that health state utility elicitation using SG could be used in Lebanon and other Arab populations in the MENA region. The large differences observed in the parameter estimates coefficients between the UK and Lebanon suggest it is important to have a local value set. However, further research is recommended to determine whether SG is feasible and acceptable in a sample in Lebanon with lower education levels, and to further generate a definitive value set for Lebanon using a representative sample of the general population.

## Figures and Tables

**Table 1:** Sociodemographic characteristics of respondents.

Characteristic	Total Sample (*N* = 124)	Test–Retest Subjects (*N* = 21)	Lebanese General Adult Population (*N* = 6,100,075) *
Mean age in years (SD)	32.45 (12.39)	29.31 (8.19)	31 **
Male/female (%)	50.8/49.2	42.9/57.1	50.2/49.8 ***
Educational level (%)			
Intermediate or secondary	14 (11.3)	2 (9.5)	36.8 ****
Degree and above	110 (88.7)	19 (90.5)	13.4%
Marital status (%)			
Single	79 (63.7)	18 (85.7)	56% *****
Married	43 (34.7)	3 (14.3)	39%
Widowed/Divorced	2 (1.6)	-	5%
Housing type (%)			
Private	48 (38.7)	6 (28.6)	-
Rental	24(19.4)	2 (9.5)	-
Living with parents/roommates	52 (41.9)	13 (61.9)	-
Monthly household income (%)			
Less than 2,399,000 LL~1599.33 USD	20 (16.4)	5 (23.8)	-
2,400,000–3,299,000 LL~1600–2199.33 USD	15 (12.3)	3 (14.3)	-
Greater than 3,300,000 LL~2200 USD	89 (71.3)	13 (61.9)	-

* CIA Factbook, 2019; ** Mouhtadi et al., 2018; *** World Bank, 2016; **** CAS, 2004; ***** CAS, 2007.

**Table 2:** Feasibility and acceptability of the SG and ranking for the SF-6D descriptive system.

Variables	Interviewer Rating of Interviewee Participation (*n* = 126)											
	Problem in Performing Task (%)			Effort and Concentration (%)			Problem in Performing Task (%)			Effort and Concentration (%)		
	None (47.6)	Some (51.6)	A Lot (0.8)	Great (59.7)	Some (34.7)	Little (5.6)	None (58.1)	Some (41.1)	A Lot (0.8)	Great (27.4)	Some (66.1)	Little (6.5)
Interviewee self-evaluation	Ranking						SG					
Challenge level of task												
Easy (37.1%)	21.0	16.1	0.0	20.2	12.9	4.0	23.4	13.7	0.0	7.3	25.8	4.0
Neutral (37.9%)	13.7	23.4	0.8	24.2	12.9	0.8	20.2	16.9	0.8	10.5	25.8	1.6
Difficult (25.0%)	12.9	12.1	0.0	15.3	8.9	0.8	14.5	10.5	0.0	9.7	14.5	0.8
Tried best to answer												
Yes (92.7)	45.2	46.8	0.8	56.5	31.5	4.8	55.6	36.3	0.8	25.8	61.3	5.6
No (7.3)	2.4	4.8	0.0	3.2	3.2	0.8	2.4	4.8	0.0	1.6	4.8	0.8
Number of dimensions considered in SG												
None (13.7)	7.3	6.5	0.0	8.1	5.6	0.0	7.3	6.5	0.0	4.0	8.9	0.8
One (2.4)	0.8	1.6	0.0	0.8	1.6	0.0	1.6	0.8	0.0	0.0	2.4	0.0
Two (33.9)	16.9	16.9	0.0	22.6	10.5	0.8	22.6	11.3	0.0	6.5	26.6	0.8
≥Three (50.0)	22.6	26.6	0.8	28.2	16.9	4.8	26.6	22.6	0.8	16.9	28.2	4.8
Quality of answers												
Very good (33.9%)	16.1	17.7	0.0	19.4	12.1	2.4	20.2	13.7	0.0	8.1	22.6	3.2
Good (51.6%)	25.0	25.8	0.8	31.5	17.7	2.4	29.8	21.8	0.0	15.3	33.9	2.4
Average (14.5%)	6.5	8.1	0.0	8.9	4.8	0.8	8.1	5.6	0.8	4.0	9.7	0.8
Felt bored or irritated												
Yes (21.0)	1.6	2.4	0.0	1.6	2.4	1.6	0.0	1.6	2.4	2.4	1.6	0.0
No (79.0)	46.0	49.2	0.8	46.0	57.3	33.1	5.6	56.5	38.7	25.0	64.5	6.5

SG: Standard gamble; SF-6D: Short form 6 dimensions.

**Table 3:** Descriptive statistics for the 50 SF-6D health state valuations comparing Lebanon and the UK.

	Lebanon						United Kingdom					
Health State	Mean	S.D.	Minimum	Maximum	Median	*N*	Mean	S.D.	Minimum	Maximum	Median	*N*
111,621	0.824	0.114	0.440	0.950	0.850	18	0.620	0.414	−0.060	0.990	0.845	10
113,411	0.854	0.098	0.550	0.960	0.885	18	0.597	0.363	−0.140	0.980	0.610	12
115,653	0.730	0.152	0.370	0.920	0.760	18	0.581	0.273	0.100	0.980	0.590	8
121,212	0.842	0.102	0.460	0.910	0.855	18	0.783	0.235	0.280	0.970	0.783	7
122,233	0.869	0.044	0.760	0.940	0.880	17	0.827	0.233	0.140	1.000	0.905	14
122,425	0.758	0.173	0.370	0.930	0.820	17	0.657	0.357	0.100	1.000	0.855	10
124,125	0.848	0.132	0.370	0.960	0.890	18	-	-	-	-	-	-
131,542	0.828	0.102	0.520	0.950	0.880	18	0.424	0.414	−0.660	0.960	0.450	17
132,524	0.763	0.168	0.370	0.940	0.820	18	0.580	0.352	0.000	1.000	0.615	8
133,132	0.858	0.046	0.760	0.940	0.855	18	0.569	0.364	0.000	1.000	0.670	11
135,312	0.756	0.176	0.370	0.910	0.820	18	-	-	-	-	-	-
142,154	0.791	0.159	0.370	0.920	0.850	17	0.513	0.378	0.280	0.950	0.310	10
144,341	0.742	0.194	0.190	0.920	0.820	17	0.727	0.247	0.120	0.990	0.825	30
211,111	0.890	0.042	0.820	0.960	0.900	18	0.778	0.276	0.190	1.000	0.905	10
212,145	0.785	0.152	0.370	0.950	0.830	18	-	-	-	-	-	-
213,323	0.783	0.156	0.360	0.940	0.820	18	0.743	0.255	0.120	0.980	0.790	12
221,452	0.824	0.079	0.640	0.940	0.820	18	-	-	-	-	-	-
224,612	0.646	0.170	0.360	0.900	0.640	18	0.540	0.380	−0.240	0.880	0.670	9
232,111	0.858	0.062	0.700	0.940	0.880	17	0.759	0.359	0.000	1.000	0.960	9
235,224	0.767	0.164	0.280	0.910	0.835	17	0.468	0.307	0.100	0.990	0.430	11
241,531	0.785	0.179	0.190	0.920	0.845	18	0.753	0.237	0.280	0.990	0.880	17
312,332	0.864	0.047	0.720	0.950	0.880	18	0.778	0.267	0.190	1.000	0.910	12
315,515	0.698	0.195	0.330	0.940	0.745	18	0.559	0.254	0.190	0.970	0.550	15
321,122	0.858	0.062	0.720	0.940	0.850	18	0.757	0.248	0.190	0.990	0.850	17
323,644	0.571	0.200	0.190	0.900	0.575	18	0.397	0.309	0.100	0.990	0.290	10
332,411	0.844	0.062	0.720	0.940	0.860	17	0.770	0.269	0.190	1.000	0.835	12
334,251	0.734	0.149	0.370	0.900	0.800	17	-	-	-	-	-	-
341,123	0.831	0.170	0.190	0.960	0.880	18	0.757	0.313	0.100	0.990	0.920	10
412,152	0.793	0.129	0.460	0.950	0.850	18	0.501	0.284	0.100	0.930	0.590	10
414,522	0.755	0.172	0.330	0.940	0.810	18	0.541	0.390	−0.010	1.000	0.570	11
421,314	0.811	0.090	0.630	0.940	0.820	18	0.713	0.341	0.100	1.000	0.845	12
425,131	0.658	0.188	0.360	0.900	0.715	18	-	-	-	-	-	-
431,443	0.824	0.104	0.460	0.920	0.850	17	0.613	0.384	0.000	1.000	0.805	12
432,621	0.743	0.150	0.370	0.900	0.820	17	-	-	-	-	-	-
443,215	0.731	0.242	0.190	0.920	0.840	18	0.673	0.345	−0.060	1.000	0.805	12
511,114	0.858	0.059	0.720	0.950	0.880	18	0.604	0.316	0.100	1.000	0.590	13
512,242	0.603	0.230	0.190	0.940	0.550	18	0.705	0.188	0.250	0.910	0.750	11
522,321	0.777	0.163	0.280	0.940	0.840	18	0.675	0.317	0.120	0.990	0.700	11
523,551	0.607	0.231	0.190	0.900	0.678	18	-	-	-	-	-	-
531,635	0.786	0.118	0.510	0.920	0.820	17	0.439	0.950	−0.850	0.950	0.450	14
534,113	0.723	0.191	0.280	0.930	0.800	17	-	-	-	-	-	-
545,422	0.700	0.248	0.190	0.920	0.825	18	0.604	0.325	0.100	0.990	0.620	9
611,221	0.821	0.087	0.580	0.950	0.830	18	-	-	-	-	-	-
614,434	0.561	0.265	0.190	0.940	0.530	18	0.652	0.292	0.110	0.960	0.710	13
622,513	0.707	0.171	0.360	0.920	0.720	18	0.567	0.368	0.000	1.000	0.640	13
625,141	0.510	0.236	0.190	0.900	0.505	18	0.703	0.312	0.140	0.990	0.860	10
631,355	0.741	0.152	0.370	0.920	0.760	17	0.657	0.300	0.100	0.980	0.700	15
633,122	0.714	0.253	0.190	0.930	0.800	17	0.466	0.353	0.000	0.910	0.470	8
642,612	0.685	0.245	0.190	0.920	0.775	18	0.484	0.397	−0.280	1.000	0.675	18
645,655	0.322	0.190	0.100	0.750	0.300	124	0.213	0.428	−0.980	0.980	0.050	622

SD: Standard deviation; RE: Random effect.

**Table 4:** Mean and individual level models for Lebanon and UK.

Parameter	RE		Mean	
	Lebanon	UK	Lebanon	UK
PF2	−0.061	−0.058	−0.056	−0.060
PF3	−0.056	−0.051	−0.058	−0.020
PF4	−0.069	−0.088	−0.071	−0.060
PF5	−0.106	−0.061	−0.106	−0.063
PF6	−0.173	−0.160	−0.168	−0.131
RL2	−0.057	−0.056	−0.049	−0.057
RL3	−0.039	−0.076	−0.004	−0.068
RL4	−0.119	−0.078	−0.049	−0.066
SF2	−0.050	−0.066	−0.063	−0.071
SF3	−0.050	−0.048	−0.106	−0.084
SF4	−0.072	−0.066	−0.122	−0.093
SF5	−0.116	−0.109	−0.162	−0.105
PAIN2	−0.013	−0.042	−0.032	−0.048
PAIN3	0.005	−0.046	−0.016	−0.034
PAIN4	−0.021	−0.055	−0.034	−0.070
PAIN5	−0.018	−0.103	−0.062	−0.107
PAIN6	−0.093	−0.178	−0.102	−0.181
MH2	−0.009	−0.043	−0.011	−0.057
MH3	−0.033	−0.055	−0.022	−0.051
MH4	−0.082	−0.115	−0.098	−0.121
MH5	−0.083	−0.125	−0.064	−0.140
VIT2	0.005	−0.040	−0.009	−0.094
VIT3	−0.010	−0.030	0.006	−0.069
VIT4	−0.025	−0.040	−0.042	−0.069
VIT5	−0.055	−0.087	−0.024	−0.106
N	992	3518	50	249
Adjusted R^2^	N/A	N/A	0.950	0.508
Inconsistencies	2	4	2	5
MAE	0.050	0.078	0.036	0.074
AE > 0.05	20	122	14	118
AE > 0.10	4	59	1	52

RE: Random effect; PF: Physical functioning; RL: Role limitation; SF: Social functioning; PAIN: Pain; MH: Mental health; VIT: Vitality; R^2^: R-squared; MAE: Mean absolute error; AE: Absolute error. Estimates shown in bold are significant at α < 0.05.
